# Intervention for early diabetic nephropathy by mesenchymal stem cells in a preclinical nonhuman primate model

**DOI:** 10.1186/s13287-019-1401-z

**Published:** 2019-12-02

**Authors:** Xingxing An, Guangneng Liao, Younan Chen, Ai Luo, Jingping Liu, Yujia Yuan, Lan Li, Lichuan Yang, Hong Wang, Fang Liu, Guang Yang, Shounan Yi, Yuanmin Li, Jingqiu Cheng, Yanrong Lu

**Affiliations:** 1 Key Laboratory of Transplant Engineering and Immunology, NHFPC, West China Hospital, Sichuan University, Chengdu, China; 2 Animal Center, West China Hospital, Sichuan University, Chengdu, China; 3 Sichuan Neo-Life Stem Cell Biotech Inc., Chengdu, China; 4 Department of Nephrology, West China Hospital, Sichuan University, Chengdu, China; 5 Department of Ultrasound, West China Hospital, Sichuan University, Chengdu, China; 6 Center for Transplant and Renal Research, Westmead Institute for Medical Research, The University of Sydney, Camperdown, NSW Australia

**Keywords:** Diabetic nephropathy, Nonhuman primate model, Mesenchymal stem cells, Inflammation, SGLT2 inhibition

## Abstract

**Background:**

Diabetic nephropathy (DN) is one of the most severe chronic diabetic complications and the main cause of end-stage renal disease. Chronic inflammation plays a key role in the development of DN. However, few treatment strategies are available; therefore, new and effective strategies to ameliorate DN at the early stage must be identified.

**Methods:**

Mesenchymal stem cells (MSCs) are characterized by anti-inflammatory and immune regulatory abilities. We developed a rhesus macaque model of DN and administered MSCs four times over 2 months. We measured blood glucose level, HbA1c, and levels of renal function parameters in the blood and urine, and cytokine levels in the kidney and blood circulatory system of rhesus macaques. Also, we analyzed the renal pathological changes of rhesus macaques. In vitro, we treated tubular epithelial cells (HK2) with 30 mmol/L glucose and 10 ng/mL human recombinant TNF-alpha (rhTNF-α) and explored the effects of MSCs on inflammation and Na^+^-glucose cotransporter 2 (SGLT2) expression in HK2.

**Results:**

We found that MSCs decreased the blood glucose level and daily insulin requirement of DN rhesus macaques. Furthermore, MSCs had a dominant function in improving renal function and decreasing SGLT2 expression on renal tubular epithelial cells. Also, renal pathological changes were ameliorated after MSC treatment. Moreover, MSCs powerfully reduced inflammation, especially decreased the level of pro-inflammatory cytokine interleukin-16 (IL-16), in the kidney and blood circulatory system.

**Conclusions:**

Our study is an important step to explore the mechanism of MSCs in ameliorating the early stage of DN, potentially through influencing SGLT2 expression and resulting in improved glycemic control and anti-inflammation. We hope these findings would provide insights for the clinical application of MSCs in DN.

**Electronic supplementary material:**

The online version of this article (10.1186/s13287-019-1401-z) contains supplementary material, which is available to authorized users.

## Background

Diabetes represents a source of worldwide health concerns, and the estimated number of people who will have diabetes by 2035 is 592 million. Diabetic nephropathy (DN), which is one of the most severe complications of diabetes [1], has been the main cause of end-stage renal disease in developed countries. Patients with type 1 diabetes typically develop DN after diabetes duration of 10 years but may be present at diagnosis of type 2 diabetes [2]. About 40% of type 2 diabetic patients develop DN [3–5]. DN is characterized by increased urinary albumin excretion, microalbuminuria, and reduced renal function indicated by an increased plasma creatinine concentration or a decreased glomerular filtration rate (GFR) [6]. DN is also an inflammatory disease, and the levels of inflammatory factors increase in patients with this disease [7].

Previously, we have established a preclinical nonhuman primate (*Macaca mulatta*) model of DN [8], which lays the foundation of this study for evaluating potential therapeutic strategies of DN. The treatments of DN mainly involve agents to ameliorate the disease. Na^+^-glucose cotransporter 2 (SGLT2) inhibitors are new and efficient drugs that have been studied in some clinical trials aiming to treat DN based on the effect of SGLT2 on glucose reabsorption in the early proximal tubule [9], which accounts for ∼ 97% of renal glucose reabsorption under normal glycemic conditions. Based on evidence showing the restoration of tubuloglomerular feedback via the direct dilation of the afferent arteriole and indirect induction of vasoconstriction of the efferent arteriole, SGLT2 inhibitors increase the GFR [10]. Although the SGLT2 inhibitor has been shown to inhibit renal glucose reabsorption, new mechanisms of this drug, such as its influence on inflammation, are thought-provoking and require further investigation [11, 12], especially for DN, which is a low inflammation disease.

Mesenchymal stem cells (MSCs) represent an attractive regenerative therapy [13] and were first identified and isolated from bone marrow, and they are characterized by the ability to differentiate into tissues of mesodermal origin [14]. Currently, MSCs can be isolated from a variety of organisms and tissues [15–17]. Additionally, MSCs present anti-inflammatory and immune regulatory abilities [18]. A large number of studies have investigated the role of MSCs in many diseases, and they have been clinically approved for the treatment of graft-versus-host disease; moreover, 379 clinical trials of MSCs associated with diabetes (3 trials for chronic kidney disease) are registered on ClinicalTrials.gov, and they have been used to treat autoimmune diseases [19, 20]. Many animal experiment studies have applied MSCs for retinopathy [21], myocardial infarction [22], diabetes [23], and DN in rats and mice [24, 25]. Almost all studies of MSCs on DN have been conducted in rat or mouse models, which are genetically heterogeneous compared to humans, and the mechanisms of MSCs are not clear; therefore, studies in nonhuman primates are required because they could offer significant preclinical evidence of MSC applications.

Based on the abundant source, anti-inflammatory and immune regulatory features of MSCs, we administered MSCs in our rhesus macaque model of DN to explore the effects and mechanisms of MSCs on DN and answer the following questions: What are the interactions between MSCs and SGLT2 protein? Would MSC influence on glycemic control of DN rhesus macaques? Does MSCs transplantation result in SGLT2 inhibition thus contributing to reduced inflammation in kidneys? These questions are though-provoking and worthy of investigation.

## Material and methods

### Recombinant proteins and antibodies

Human recombinant TNF-alpha (rhTNF-α) (R&D Systems) was used at a final concentration of 10 ng/mL. All primary and secondary antibodies and their dilutions are described in Additional file 1: Table S1.

### Rhesus macaques

Fifteen adult healthy rhesus macaques (male, aged 3–5 years) were obtained from Chengdu Ping’an Experimental Animal Reproduction Center ((license no.: SCXK (CHUAN) 2014-013, Chengdu, China). The use and care of animals were in accordance with the guidelines of the Institutional Animal Care and Use Committee of Experimental Animal Center, West China Hospital, Sichuan University (Chengdu, China) (Protocol: 2014004A), which have been approved by the Association for the Assessment and Accreditation of Laboratory Animal Care International (AAALAC). Rhesus macaques were randomly divided into a normal control group (*n* = 3), which was fed a standard diet twice per day, and an experimental group (*n* = 12), which were administered a single high dose of streptozotocin (STZ, 80 mg/kg) (Sigma-Aldrich) intravenously to induce diabetes as previously described [26]. Insulin was used to maintain the FBG level at 15–20 mmol/L in all diabetic rhesus macaques. To develop DN, a diet containing 10 g of salt and 60 g of peanuts was administered to diabetic rhesus macaques for at least 2 years as previously described [8]. Subsequently, rhesus macaques with DN were randomly divided into a normal saline-treated group (DN + NS, *n* = 4) and a MSC-treated group (DN + MSCs, *n* = 8); two DN rhesus macaques were euthanized for analyzing MSCs in organs at 1 week after MSC treatment. During the entire experiment, death or severe disease did not occur in any of the rhesus macaques.

### Human umbilical cord-derived MSCs

Human umbilical cords were donated by women who underwent cesarean sections. Informed consent was obtained. Human umbilical cord-derived MSCs were freshly isolated from a single donor, characterized by MSC markers with flow cytometry, and established in terms of phenotypic profile, colony-forming potential, and differentiation potential to chondroblasts, osteoblasts, and adipocytes at Sichuan Stem Cell Bank, Chengdu, China (Additional file 1: Figure S1). In addition, viral factors, pathogenic organisms, and endotoxin levels of MSCs were monitored (Additional file 1: Table S2). MSCs were cultured with serum-free medium (Stem cell 05420MesenCultTM-XF Medium, StemRD). Cells between passages 3 and 5 were used for all experiments [27].

### Delivery of MSCs

Freshly isolated and identified MSCs from a single donor were suspended in 100 mL normal saline (NS) and delivered at a density of 2 × 10^6^ cells/kg to one DN rhesus macaque at an infusion rate of 45–50 drops/min. And each rhesus macaque was treated with MSC derived from a different umbilical cord. It took about 45–60 min to complete the infusion. A total four times of MSC transplantation were performed during 2 months. MSCs were labeled with 1 μg/mL CM-Dil cell tracker (Invitrogen), incubated at 37 °C for 5 min and 4 °C for 15 min, washed three times, and suspended in NS at the final MSC transplantation.

### Blood analysis

Whole blood for routine examination and serum for biochemistry analysis were monitored every 3 months during the establishment of rhesus macaque model of DN, 1 day before MSC transplantation, and different months after MSC transplantation of rhesus macaque models. The levels of glucose, serum creatinine (Scr), blood urea nitrogen (BUN), and estimated glomerular filtration rate (eGFR) were analyzed using an automatic biochemistry analyzer (Hitachi, Tokyo, Japan).

### Urine analysis

Urine samples were collected every 3 months after STZ injection and 1 month after MSC transplantation of rhesus macaques in individual primate metabolic cages. The levels of urinary microalbumin and urinary creatinine were assayed by a protein assay kit (Bio-Rad). Urinary microalbumin excretion was defined as the ratio of urinary microalbumin to creatinine (UACR) in a range of 2.5–25 mg/mmol [6].

### Renal histopathology

Percutaneous ultrasound-guided renal biopsy was performed in rhesus macaques before and 1 month after MSC or NS infusion. Hematoxylin and eosin (H&E), Masson’s trichrome (Masson), and periodic acid Schiff (PAS) staining and transmission electron microscopy (TEM) analyses of renal tissues were examined by two expert renal pathologists who were blinded to the experiments. Four fields of each section, and four sections per rhesus macaque were observed and quantified. Glomerular basement membrane thickness was determined by the orthogonal intercept method [28]. The renal histological changes for glomerulosclerosis, tubular dilation, mesangial matrix deposition, and interstitial fibrosis were evaluated semiquantitatively by a scoring system of 0–3, where 0 = no change, 1 = mild changes, 2 = moderate changes, and 3 = severe changes [29].

### Immunohistochemistry

Renal sections were fixed with 4% paraformaldehyde, incubated with primary antibodies at 4 °C overnight and secondary antibody at 37 °C for 2 h, washed three times, and observed under a light microscope. Four fields of each section, and four sections per rhesus macaque were observed and quantified. The results of immunohistochemistry were analyzed by Image Pro Plus software.

### Cell culture

Human proximal tubular cells (HK2) were obtained from ATCC (CRL-2190). To mimic the hyperglycemia and low-state inflammation environment, 30 mmol/L glucose with 10 ng/mL rhTNF-α (GT) was used. GT-treated HK2 cells were cocultured with MSCs in a Transwell plate at a 5:1 ratio (HK2: MSCs) for subsequent experiments. Each group had three replicates in one experiment, and all experiments in vitro were independently repeated three times unless indicated otherwise.

### RNA extraction and real-time qPCR

RNA was extracted from renal biopsies and HK2 cells using a Promega SV Total RNA Isolation System (Promega) according to the manufacturer’s instructions. Primers were synthesized by Sangon Biotech (Shanghai, China). β-actin was used as the internal reference gene, and all primer sequences are listed in Additional file 1: Table S3. Real-time qPCR was carried out, and the relative fold change (2^−ΔΔCT^) was calculated from the obtained CT values.

### Magnetic Luminex assay

The levels of cytokines in rhesus macaque serum were measured by a Quantibody® Non-Human Primate Cytokine Array 1 (Raybiotech, Guangzhou, China).

### Glucose uptake

The level of glucose in HK2 cell culture medium was measured by the glucose oxidase (GOD) method. Intracellular glucose levels were represented by ^18^F radioactivity in HK2 cells incubated with [^18^F]-FDG (1 μCi/mL; 0.037 MBq/mL) in DMEM either in the presence or absence of high glucose for 2 h. ^18^F radioactivity was measured on a γ counter (Wallac).

### Statistical analysis

Data were presented as the mean (± s.e.m.). Statistical analysis was performed using GraphPad 8.0 software. Statistical evaluation of two groups was performed using a Student *t* test or Mann–Whitney *U* test if data were not normally distributed. A two-way ANOVA was used to compare the differences between the groups of rhesus macaques and data collected at different time points. And the statistical analysis was corrected for repeated measures when comparing multiple measurements within subjects. A *p* value less than 0.05 was considered statistically significant.

## Results

### High-salt and high-fat diet induced early stage of DN in rhesus macaques

**Fig. 1:**
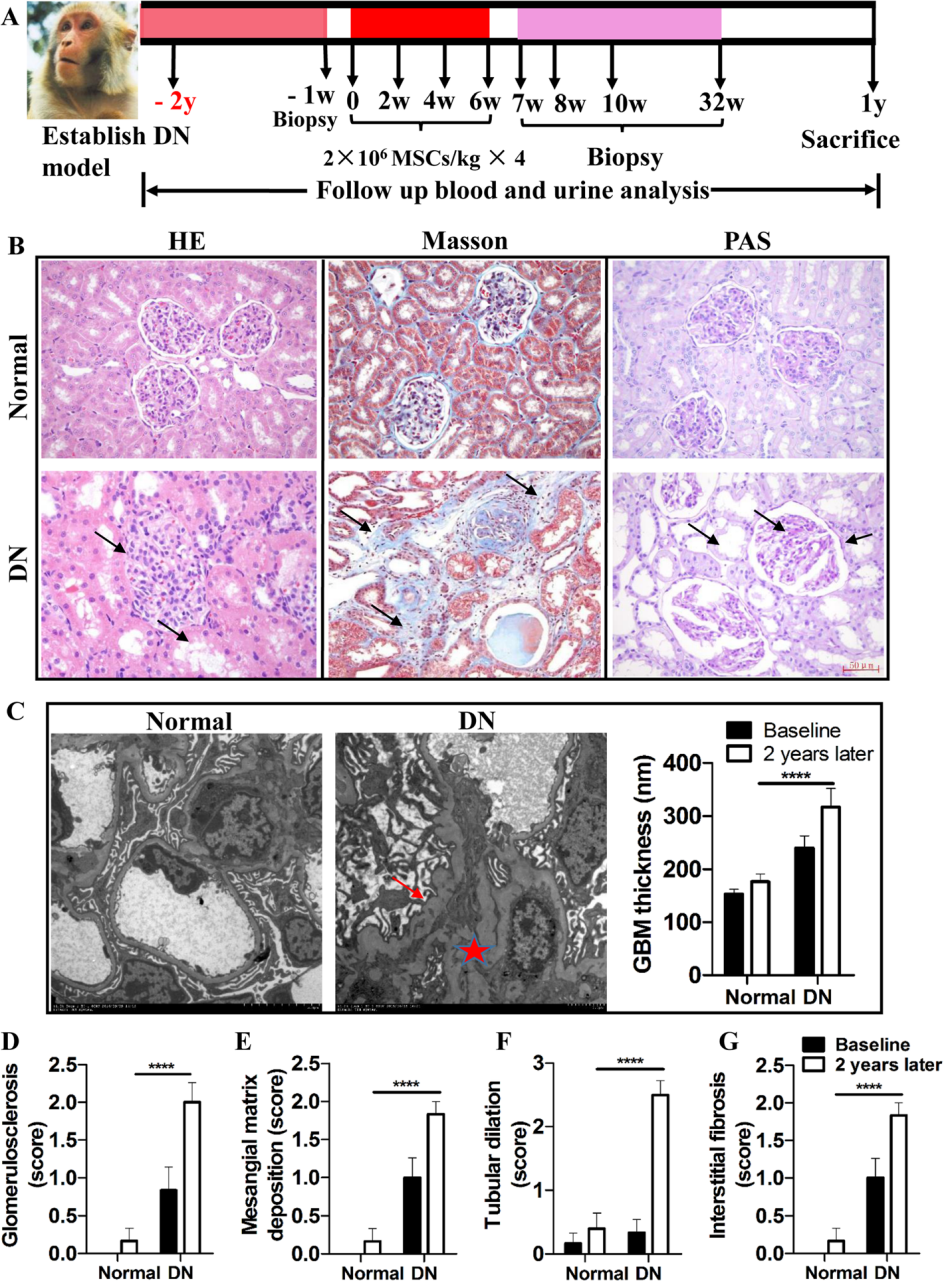
Rhesus macaques presenting an early stage of DN. **a** Timeline of the study. **b** Histological staining of renal tissues. Glomerular hypertrophy, tubular dilatation, and peritubular interstitial fibrosis were indicated by arrows. **c** TEM of renal tissues. Thickened glomerular basement membranes are indicated by red arrow and asterisk. **d**–**g** Quantified renal histological indices at baseline and 2 years later. GBM: glomerular basement membrane; Normal: normal and nondiabetic rhesus macaques; DN: rhesus macaques with diabetic nephropathy. Scale bar = 50 μm. Magnification of TEM: 1.2 k. Each bar represents the mean ± s.e.m. Four fields of each section, four sections per rhesus macaque were observed and quantified, and the number of rhesus macaques analyzed in each group: Normal (*n* = 3), DN (*n* = 12). ****p < 0.0001: normal group versus DN group after 2 years

The early stage of DN rhesus macaque model was established based on previous research in our laboratory [8]. Basal characteristics of all groups, including levels of biochemical parameters and quantified renal histological indices, are described in Table 1. Renal histological analyses of the kidney tissues, including H&E, Masson, and PAS staining (Fig. 1b), showed glomerulus hypotrophy, tubular dilatation, tubule collapse with the obliteration of the lumen, peritubular interstitial fibrosis, and thickened tubular basal lamina. TEM (Fig. 1c) showed increased mesangial matrix and thickened glomerular basement membranes (GBMs) in diabetic rhesus macaques after 2 years of a high-salt and high-fat diet.

**Table 1:** The baseline characteristics of rhesus macaques

Group			Normal (*n*=3)				DN+NS (*n*=4)							DN+MSCs (*n*=8)				
ID	05145	09388	10475	Mean (± s.e.m.)	05593	05265	06145	09475	Mean (± s.e.m.)	04053	05539	06431	07343	09011	10091	10053	10207	Mean (± s.e.m.)
Gender	Male	Male	Male		Male	Male	Male	Male		Male	Male	Male	Male	Male	Male	Male	Male	
Body weight (Kg)	4.5	4.9	4.7	4.7±0.2	5	5.1	4.9	5	5±0.08	4.7	5.1	5.3	4.6	4.9	4.7	4.8	4.4	4.81±0.29
CREA (μmol/L)	46.9	62.6	49.4	52.97±8.43	101	113.4	122.9	105.7	110.75±9.58 *	89.2	90.6	84.7	107.8	115.1	97.9	99.1	109.2	99.2±10.77 *
BUN (mmol/L)	4.74	5.17	4.23	4.71±0.47	12.19	9.43	12.27	15.42	12.33±2.45 *	8.66	10.92	9.28	12.4	8.86	11.5	10.1	10.37	10.26±1.31 *
Uric Acid (μmol/L)	1	1	1	1	2	5	3	1	2.75±1.71	3	4	4	3	4	4	3	3	3.5±0.53 *
LDL-C (mmol/L)	1.55	1.72	1.51	1.59±0.11	3.76	5.18	2.11	3.33	3.56±1.27	2.48	2.1	3.74	6.01	5.04	4.92	3.17	3.2	3.83±1.37 *
HDL-C (mmol/L)	2.34	1.85	2.04	2.07±0.25	1.66	2.12	1.21	1.82	1.7±0.38	1.07	1.48	1.71	2.5	1.87	2.04	1.59	1.95	1.78±0.42
CHOL (mmol/l)	3.63	3.75	3.48	3.62±0.14	6.64	8.13	3.73	4.76	5.82±1.96	4.97	3.4	4.9	8.17	6.88	7.76	5.24	5.05	5.8±1.64
Triglycerides (mmol/L)	0.57	0.4	0.6	0.52±0.11	0.66	2.75	0.85	1.25	1.38±0.95 *	0.81	1.56	0.44	1.99	1.63	1.44	1.03	1.5	1.3±0.5 *
FBG (mmol/L)	6.3	5.9	5.8	6±0.26	19.71	19.51	18.04	20.22	19.37±0.94 *	18.86	20.08	17.45	19.92	20.43	18.75	19.43	19.36	19.29±0.94 *
HbA1c (%)	5.7	6	5.9	5.86±0.15	11.5	10.7	10.8	11	11±0.36 *	9.5	10.2	9.3	10.3	8.4	11.5	11	10.4	10.08±0.99 *
Daily insulin (U)	0	0	0	0	1.5	1.2	1	1.5	1.3±0.25 *	1	1.2	1	1.5	1.2	1	1.5	1.2	1.2±0.21 *
Urinary microalbumin (mg/L)	0.03	0.75	1.21	0.75±0.45	11.3	10.9	16.2	12.8	12.8±2.41 *	21.5	23	20.72	17.82	13.2	9.59	12.7	10.9	16.18±5.22 *
Urinary creatinine (μmol/L)	1451	1352	1596	1466.33±122.72	1729	1525	2067	1994	1828.75±249.2	1427	1904	2414	2621	1081	1100	1025	989	1570.13±659.39
Glomerulosclerosis (score)	0	0	0	0	2	2	2	2	2±0 *	2	2	2	2	2	2	2	2	2±0 *
Mesangial matrix deposition (score)	0	0	0	0	1	2	1	2	1±0.59 *	1	2	2	1	2	2	1	2	1.63±0.52 *
Tubular dilation (score)	0	0	0	0	2	3	2	2	2.25±0.5 *	2	3	2	3	3	3	3	3	2.75±0.46 *
Interstitial fibrosis (score)	0	0	0	0	2	2	2	1	1.75±0.5 *	2	1	2	2	2	2	2	2	1.88±0.35 *

*CREA* serum creatinine, *BUN* blood urea nitrogen, *LDL-C* low-density lipoprotein, *HDL-C* high-density lipoprotein, *CHOL* cholesterol, *FBG* fasting blood glucose, *HbA1c* glycosylated hemoglobin. Score of renal histological changes: 0 = no change, 1 = mild changes, 2 = moderate changes, and 3 = severe changes

*p < 0.05: DN+NS vs. Normal, DN+MSCs vs. Normal

### Rhesus macaques immunologically tolerated human umbilical cord-derived MSCs

To evaluate whether rhesus macaques tolerate xenotransplantation of human MSCs, we tested their immune cells in peripheral blood, including the numbers of lymphocytes and monocytes, and the ratio of CD4+/CD8+ T cells (Additional file 1: Figure S2). We did not observe any prominent changes in the immune system of rhesus macaque after human umbilical cord-derived MSC infusion. Also, the weight and vital signs of rhesus macaques were stable after MSC transplantation. These data indicated the safety of xenotransplantation to rhesus macaques.

### Homing of MSCs to kidneys

**Fig. 2:**
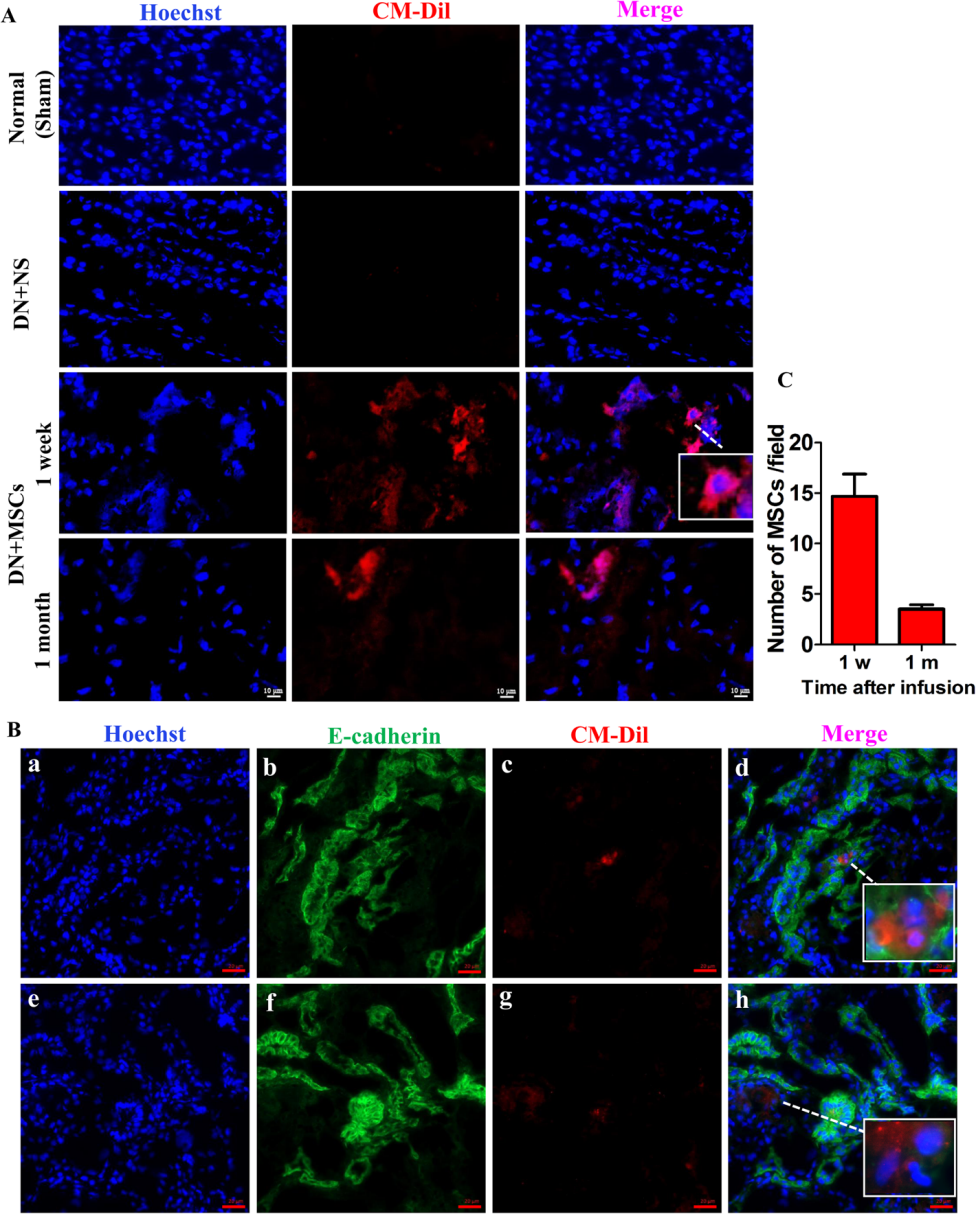
Fluorescence microscopy of MSCs in rhesus macaques. MSCs were labeled by CM-Dil (red fluorescence) before infusion to rhesus macaques. **a** Renal biopsies at 1 week and 1 month after MSC transplantation. **b** Immunostaining of kidney tissues at 1 month after MSC transplantation. **c** Quantification of number of MSCs per field at 1 week and 1 month after MSCs infusion. MSCs were labeled by CM-Dil (red), tubular epithelial cells were stained by E-cadherin (green), and cell nuclei were stained by Hoechst (blue). Scale bar of **a** = 10 μm and scale bar of **b** = 20 μm. Each bar represents the mean ± s.e.m. Four fields of each section, four sections per rhesus macaque were observed and quantified, and the number of rhesus macaques was analyzed in each group: Normal (*n* = 3), DN + NS (*n* = 4), DN + MSCs (*n* = 8)

It is necessary to determine where MSCs migrate after MSC transplantation in rhesus macaques. One week after the final transplantation of CM-Dil-labeled MSCs, two DN rhesus macaques were sacrificed and analyzed for red fluorescence in organs. We observed that the strongest fluorescence signal was in the lung, followed by the kidney (Additional file 1: Figure S3). After confirming the successful migration of MSCs to the kidney, we explored the duration that the infused MSCs remained in the kidney. One week and one month after the last injection of MSCs, we performed percutaneous renal biopsy of all rhesus macaques and observed renal sections under fluorescence microscopy. One week after MSC transplantation, we observed red fluorescence in the cytoplasm and intact cell membrane, whereas fluorescence was not observed in the renal tissue from normal or normal saline-injected rhesus macaques. One month after MSC transplantation, red fluorescence was still observed in the renal tissue, which especially arranged along tubular epithelial cells (Fig. 2A). Due to the lack of specific markers of MSCs, we used a marker of epithelial cells (E-cadherin) to help determine whether the red fluorescence was from CM-Dil-labeled MSCs and not from the resident cells in the kidney. Interestingly, MSCs were observed around tubular epithelial cells (E-cadherin positive) (Fig. 2B). Notably, the number of MSCs per field dropped significantly between 1 week and 1 month after MSC infusion (Fig. 2C). These data lay the foundation of further research, such as that to explore the effects of MSCs on tubular epithelial cells.

### MSCs improved glycemic control and renal function of rhesus macaques

**Fig. 3:**
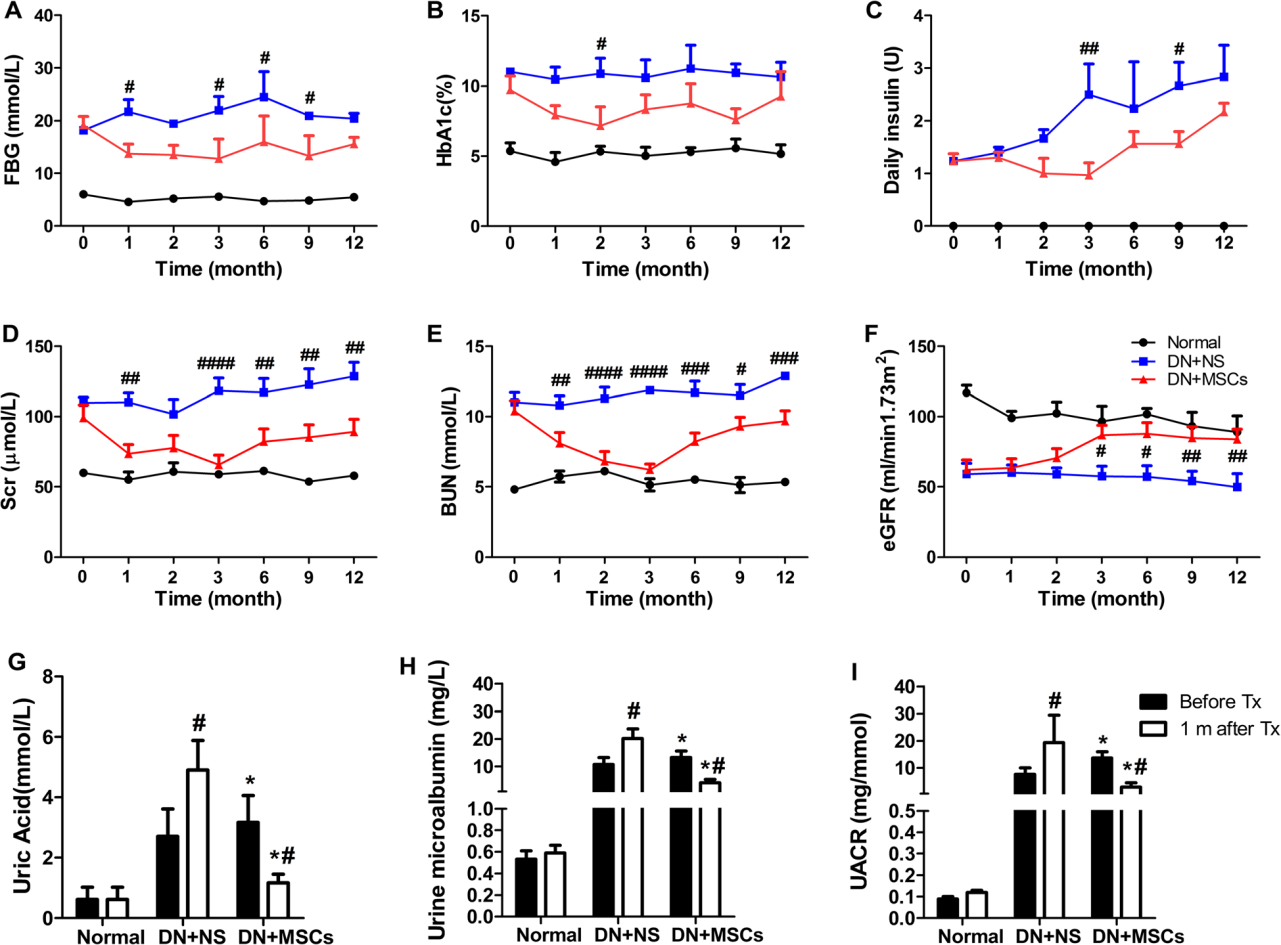
Protective effects of MSCs on rhesus macaques. Parameters related to glycemic control and renal function were measured and analyzed before (month 0), during (months 1, 2), and after (months 3, 6, 9, 12) normal saline or MSC infusion. **a** Fasting blood glucose (FBG), **b** HbA1c, **c** daily required insulin dose, **d** Levels of serum creatinine (Scr), **e** blood urea nitrogen (BUN), **f** estimated glomerular filtration rate (eGFR), **g** uric acid, **h** urine microalbumin, and **i** ratio of urinary microalbumin to creatinine (UACR) were analyzed. NS: normal saline; MSCs: mesenchymal stem cells. Each bar represents the mean ± s.e.m. Normal (*n* = 3), DN + NS (*n* = 4), DN + MSCs (*n* = 6). *p < 0.05: before versus after in DN + MSC group and DN + MSC group versus normal group. #*p* < 0.05, ##*p* < 0.01, ###*p* < 0.001, ####*p* < 0.0001: DN + NS group versus DN + MSC group after NS or MSC transplantation

After confirming that MSCs could migrate to the kidney, we then explored the effects of MSCs on glycemic control and renal function of rhesus macaques. Although we used insulin to maintain a proper blood glucose level (15–20 mmol/L) of rhesus macaques, it was necessary to assess the effect of MSC on glycemic control which is always a primary concern of treatments of diabetic complications. Interestingly, we observed that the average level of fasting blood glucose (Fig. 3a) and HbA1c (Fig. 3b), and daily requirement of insulin (Fig. 3c) in rhesus macaques with MSC treatment were significantly lower than those with normal saline infusion. During MSC transplantation, the levels of Scr (Fig. 3d) and BUN (Fig. 3e) decreased. However, the protective effect of MSCs did not persist over time because we observed that the levels of Scr and BUN tended to increase again after only one course of MSC treatment in rhesus macaques. The level of eGFR increased after MSC transplantation but decreased after NS infusion (Fig. 3f). The levels of UA (Fig. 3g), urine microalbumin (Fig. 3h), and UACR (Fig. 3i) declined significantly after MSC transplantation and were much lower than those after NS infusion. These results suggested that MSCs ameliorated the progression of diabetic nephropathy in combination with insulin and indicated a possibility that the effects of MSC on improved renal function might partly be attributed to improved glycemic control.

Ultrasound is crucial for evaluating the patterns of vascularization of the kidney and contributes to the diagnosis and prediction of the early progression of chronic kidney disease [30]. Only rhesus macaques in the MSC treatment group were analyzed for renal vascularization by contrast-enhanced ultrasound (CEUS) before and after MSC treatment. We observed that the mean transit time, time to peak, and area under the descending curve decreased after MSC transplantation (Additional file 1: Figure S4), which suggested an improved renal perfusion and clearance. However, CEUS analysis should also be performed in rhesus macaques before and after normal saline infusion to enhance the evidence that improved renal perfusion was ascribed to MSC treatment.

### MSCs ameliorated renal pathological changes

**Fig. 4:**
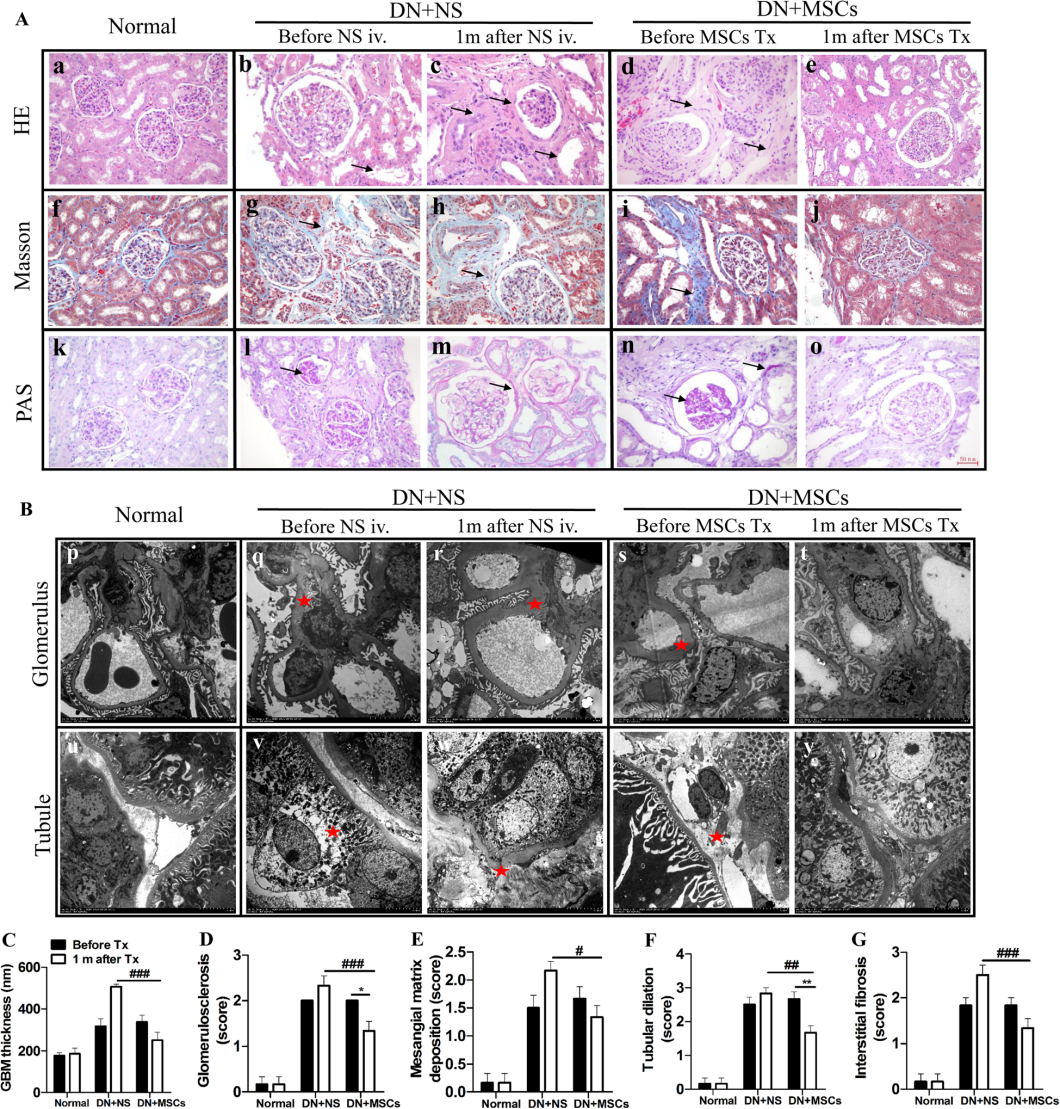
Histopathology analysis of tissues from rhesus macaques. Pathological changes were observed under a light microscope (**a**) and transmission electron microscope (**b**). Normal rhesus macaque glomerulus and tubes (a, f, k, p, and u); rhesus macaques with DN before normal saline infusion (b, g, l, q, and v) or MSC transplantation (d, i, n, s, and x). Further expanded mesangial matrix and progressive peritubular interstitial fibrosis after normal saline infusion (c, h, m, r, and w), and ameliorated glomerular basement membranes, mesangial matrix, and peritubular interstitial fibrosis after MSC transplantation (e, j, o, t, and y). The arrows and red asterisks represent pathological areas. **c**–**g** Quantified renal histological indices before and 1 month after NS or MSC transplantation. GBM: glomerular basement membrane; NS: normal saline; MSCs: mesenchymal stem cells. Scale bar of pictures in **a**: 50 μm; magnification of TEM: 1.2 k. Each bar represents the mean ± s.e.m. Four fields of each section, four sections per rhesus macaque were observed and quantified, and the number of rhesus macaques was analyzed in each group: Normal (*n* = 3), DN + NS (*n* = 4), DN + MSCs (*n* = 6). *p < 0.05, **p < 0.01: before versus after in DN + MSC group. #*p* < 0.05, ##*p* < 0.01, ###*p* < 0.001: DN + NS group versus DN + MSC group after NS or MSC transplantation

Before and 1 month after NS or MSC transplantation, percutaneous renal biopsy was conducted on rhesus macaques, and renal pathological changes were analyzed. H&E staining showed noticeable improvements of renal pathological changes in DN rhesus macaques after MSC transplantation, including amelioration of glomerular hypertrophy, tubular dilatation, and peritubular interstitial fibrosis (Fig. 4e). In contrast, the pathological damages continued after NS infusion, which were indicated by a further glomerulosclerosis and tubular dilatation (Fig. 4b, c). Masson staining showed that rhesus macaques with DN had noticeable glomerulosclerosis and tubular interstitial fibrosis (Fig. 4g, i) compared with normal rhesus macaques (Fig. 4f). PAS staining showed Bowman’s capsule hyperplasia, an expanded mesangial matrix, and an increased number of mesangial cells in rhesus macaques with DN (Fig. 4l, n). MSC was associated with a stabilization of fibrosis (Fig. 4j) and mesangial matrix expansion (Fig. 4o) in contrast to the saline-treated group in which these indices worsened (Fig. 4h, m).

TEM of biopsied kidney tissues indicated differences between normal rhesus macaques and those with DN. Specifically, with regard to glomerular structures, the loose and dense layers of GBM vanished and were replaced by one mixed thickened layer; a portion of interstitial capillary walls became thinner, discontinuous, and roughened; the number of mesangial cells increased; and the matrix was increased in rhesus macaques with DN (Fig. 4q, s) compared to that in normal rhesus macaques (Fig. 4p). Regarding the renal tubule structures, the tubular epithelia swelled and tubular interstitial fibrosis was observed in the rhesus macaques with DN (Fig. 4v, x). We observed a worsening trend of renal pathological changes after NS infusion, including that the thickened GBM became worse (Fig. 4r) and tubular interstitial fibrosis increased (Fig. 4w). In contrast, after MSC transplantation, rhesus macaques with DN showed improvements in renal structures, including thinner GBM (Fig. 4t) and reduced tubular interstitial fibrosis (Fig. 4y).

### MSCs reduced SGLT2 expression and decreased inflammation in the kidney and the whole circulatory system

**Fig. 5:**
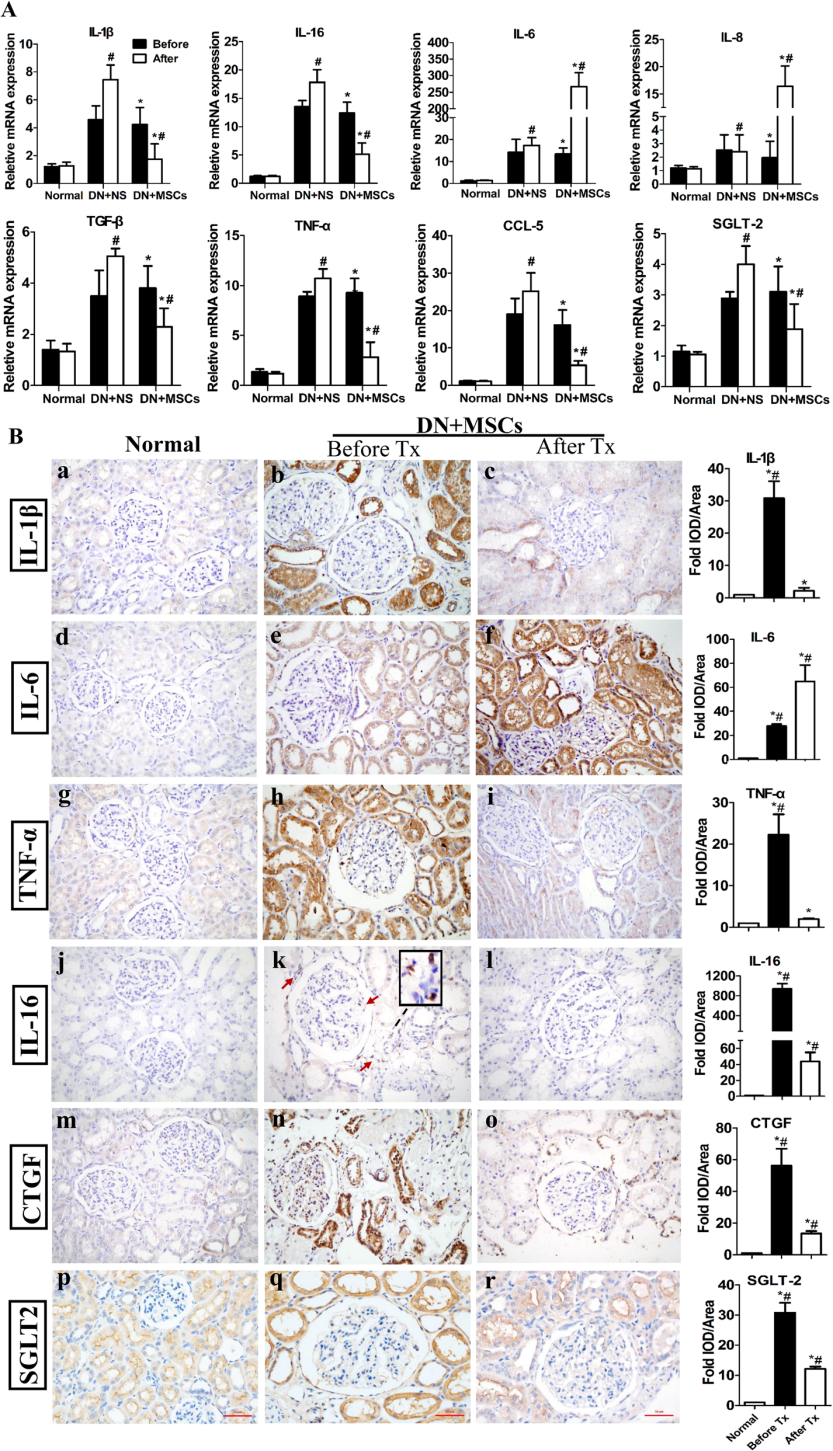
Inflammation in kidneys of rhesus macaques. RT-qPCR (**a**) and immunohistochemistry (**b**) analyses of the expression levels of inflammatory factors in renal biopsies before and after NS or MSC transplantation. NS: normal saline; MSCs: mesenchymal stem cells; Before Tx: 1 week before MSC transplantation; After Tx: 1 month after MSC transplantation. Scale bar = 50 μm. Each bar represents the mean ± s.e.m. Four fields of each section, four sections per rhesus macaque were observed and quantified, and the number of rhesus macaques was analyzed in each group: Normal (*n* = 3), DN + NS (*n* = 4), DN + MSCs (*n* = 6). *p < 0.05: before versus after in the DN + MSC group. #*p* < 0.05: DN + NS group versus DN + MSC group, before or after in the DN + MSC group versus normal group

Anti-inflammation has been reported as the most attractive feature of MSCs, which was also verified by our results. The gene expression levels of IL-1β, TNF-α, TGF-β, and CCL-5 in kidney tissues decreased. Strikingly, the levels of protein specifically expressed on tubular epithelial cells (SGLT2) and pro-inflammatory cytokine IL-16, which was released mainly by epithelial cells and immune cells, were sharply abrogated after MSC transplantation, whereas the levels of IL-6 and IL-8 increased after MSC transplantation (Fig. 5A). Consistent with the gene expression changes, the protein expression levels of IL-1β, IL-16, TNF-α, CTGF, and SGLT2 reduced after MSC transplantation, and the protein level of IL-6 increased after MSC injection (Fig. 5B).

**Fig. 6:**
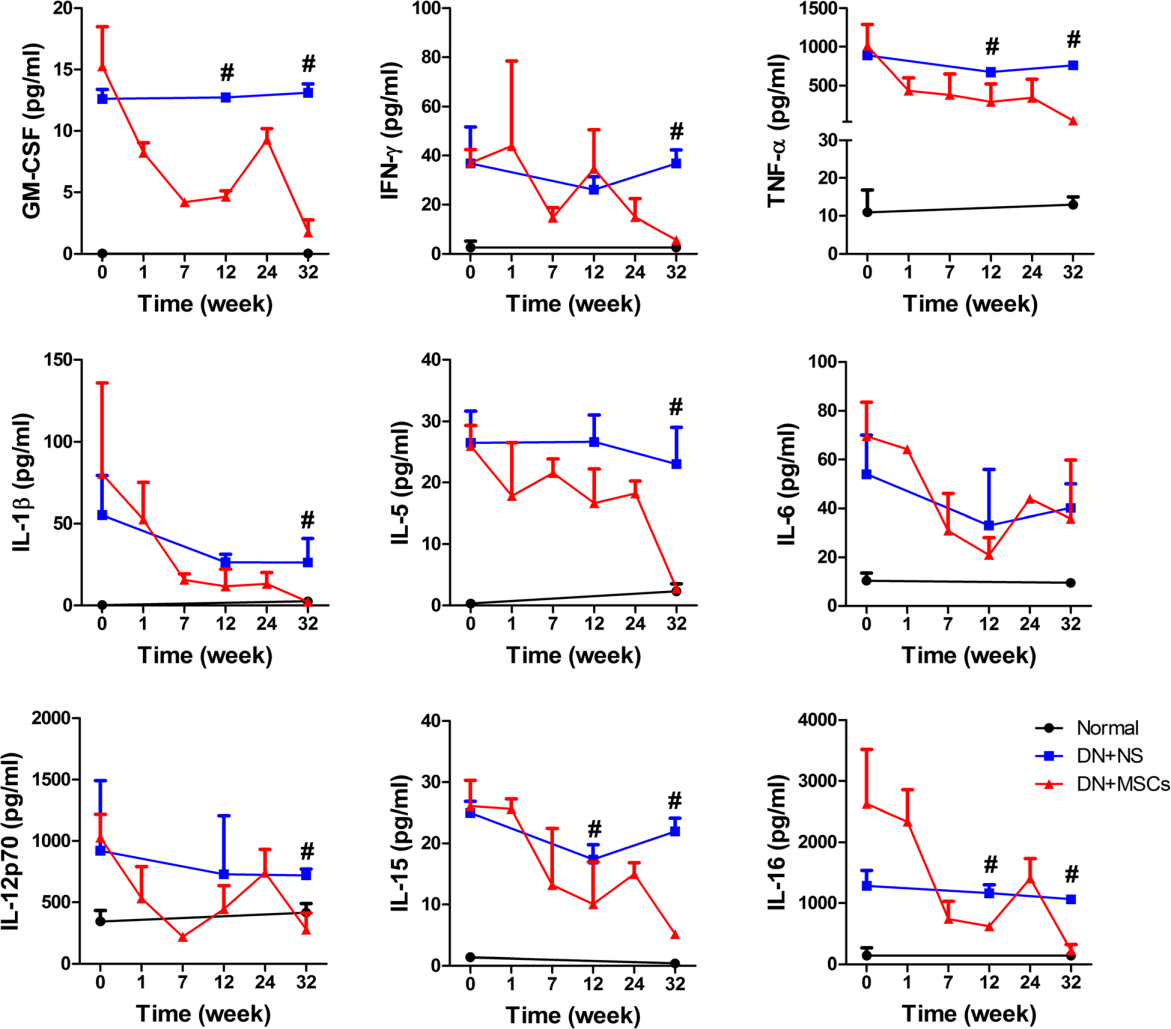
Inflammation in whole blood circulation system of rhesus macaques. Quantibody Non-Human Primate Cytokine Array of blood samples of rhesus macaques collected at different weeks after normal saline or MSC transplantation. Normal (*n* = 3), DN + NS (*n* = 4), DN + MSCs (*n* = 6). #*p* < 0.05: DN + NS group versus DN + MSC group

We further explored whether MSCs were sufficient to decrease a low state of inflammation in the whole circulatory system. We analyzed the levels of 10 inflammatory cytokines in blood serum, and the results showed that except for GM-CSF, the other cytokines decreased immediately at 1 week after the first injection of MSCs. At the seventh and 12th week, the levels of all 10 inflammatory factors were less than those before MSC transplantation. This anti-inflammatory role lasted 32 weeks (Fig. 6).

### GT-induced inflammation in HK2

**Fig. 7:**
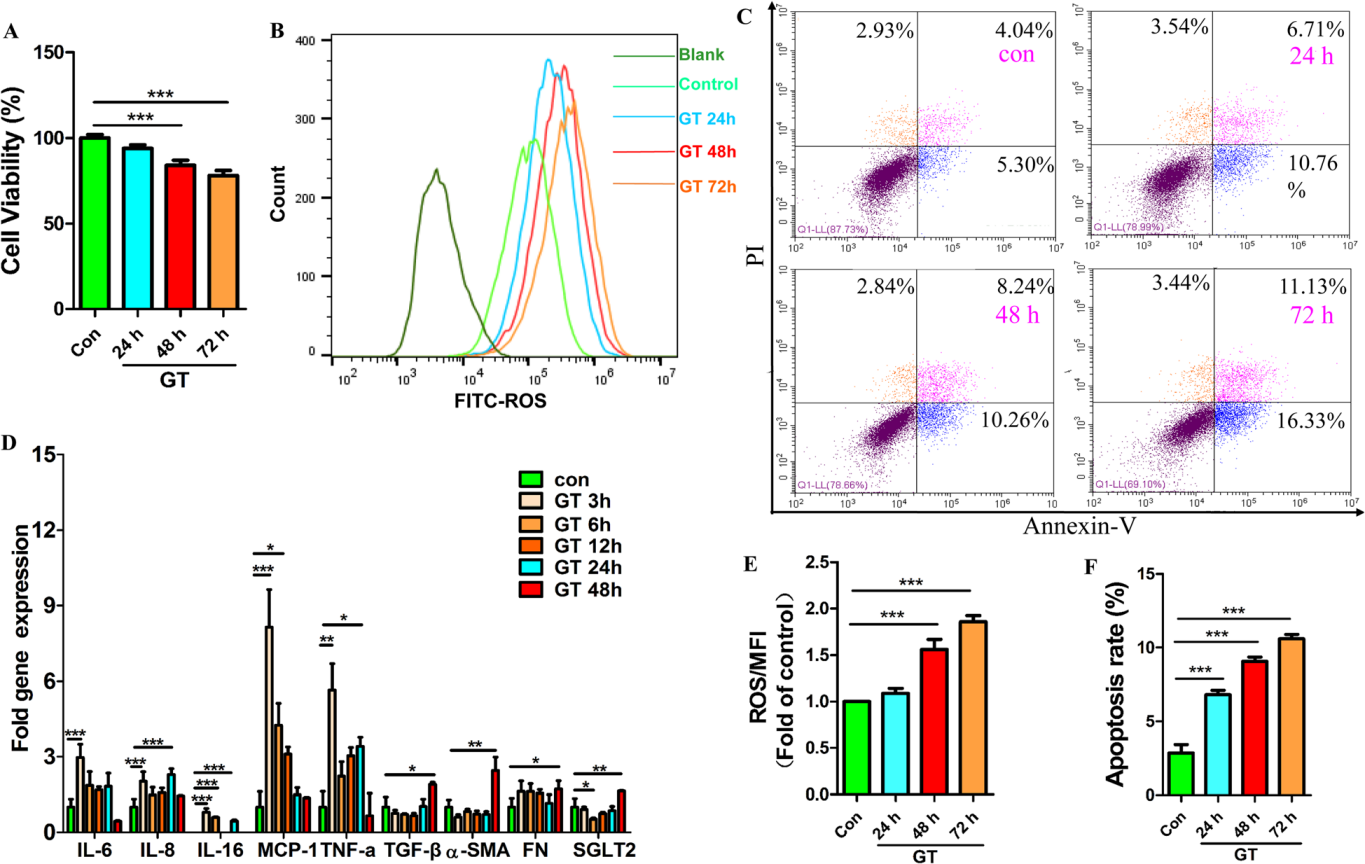
Inflammation model of HK2 cells. High glucose levels (30 mmol/L glucose) and low levels of inflammatory cytokines (10 ng/mL rhTNF-α) were used to establish the inflammation model (GT) of HK2 cells in vitro. **a** CCK-8 assay of cell viability. **b** Flow cytometry of ROS production. **c** Flow cytometry of the cell apoptosis rate. **d** qRT-PCR analysis of mRNA expression levels. **e** Quantification of ROS production. **f** Quantification of the cell apoptosis rate. Each bar represents the mean ± s.e.m. Each group had three replicates per experiment, and all experiments in vitro were independently repeated three times unless indicated otherwise. *p < 0.05: GT versus the control; **p < 0.01: GT versus the control; ***p < 0.001: GT versus the control

Previously, we established that a high level of glucose and palmitic acid induced a glucolipotoxicity damage model of endothelial cells and verified the protective effects of MSCs on glucolipotoxicity-injured endothelial cells [31]. In this study, we built a damaged model of renal epithelial cells (HK2 cells) to explore the effects of MSCs on HK2 cells. GT was used to induce damage model of HK2 cells on the basis that there was a hyperglycemic and inflammatory microenvironment in the kidney. CCK-8 assay suggested that cell viability decreased (Fig. 7a), and flow cytometry indicated that the level of ROS production (Fig. 7b) and the ratio of cell apoptosis (Fig. 7c) increased in a time-dependent manner after GT treatment. In addition, the gene expression levels of inflammatory cytokines increased as early as 3 h after GT stimulation (Fig. 7d).

### MSCs decreased SGLT2 expression and inflammation in HK2

The protein expression levels of FN, SGLT2, IL-1β, TNF-α, and phosphor-Nuclear factor-kappa B p65 (p-NF-КB p65) in HK2 cells were decreased after co-culture with MSCs for 72 h. Notably, MSCs increased the IL-6 level in GT-treated HK2 cells (Additional file 1: Figure S5a-b). Additionally, MSCs partly improved the function of epithelial cells by increasing the level of nitric oxide (Additional file 1: Figure S5c) and reduced glucose reabsorption (Additional file 1: Figure S5d).

**Fig. 8:**
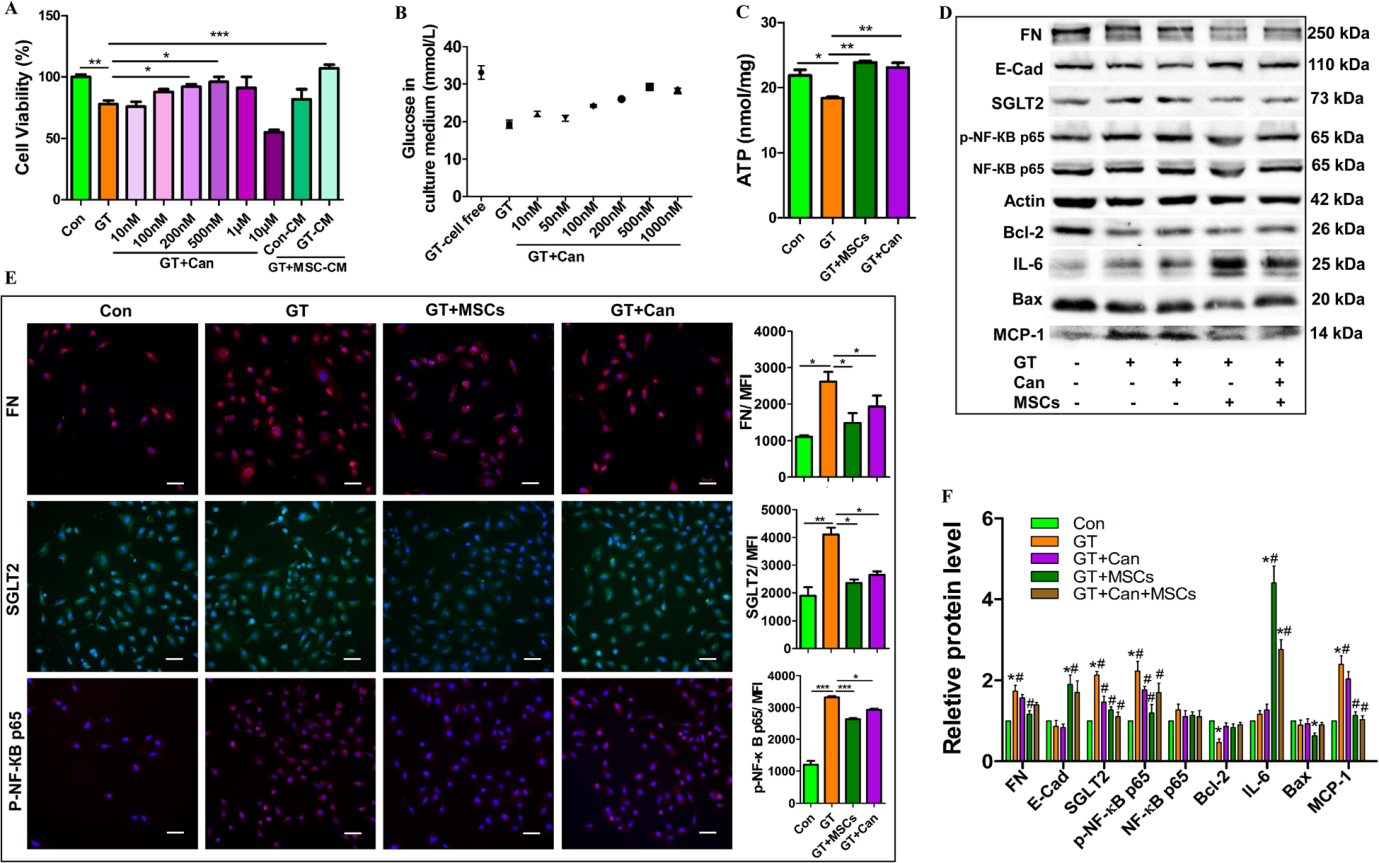
Effects of MSCs on inflammation and SGLT2 expression of HK2. HK2 cells with and without co-culture of MSCs or canagliflozin were treated with GT for 72 h. **a** The effects of canagliflozin on HK2 cell viability. **b** The levels of glucose in culture medium of HK2 cells analyzed by an oxidase method. **c** Effects of MSCs and canagliflozin on ATP level of GT-treated HK2 cells. **d** Western blot analysis of protein expression levels after MSC and canagliflozin intervention. **e** Immunostaining of FN, SGLT2, and p-NF-КB p65 in HK2 cells. **f** Quantification of Western blot analysis of protein expressions. Can: canagliflozin. Each bar represents the mean ± s.e.m., *n* ≥ 3/group. *p < 0.05; **p < 0.01; ***p < 0.001

The SGLT2 inhibitor canagliflozin has been investigated for its role in renal function [32]. In our research, canagliflozin was a positive control to explore SGLT2 inhibition. We chose a proper concentration of canagliflozin (100 nM) by analyzing its effect on cell viability (Fig. 8a) and glucose reabsorption (Fig. 8b) in GT-treated HK2 cells. SGLT2 relies on ATP to transport glucose, thus the reason for SGLT2 inhibition must be determined. Does the inhibition result from a decrease in ATP or SGLT2 expression? Hence, we measured the ATP level and found that both MSCs and canagliflozin increased the ATP level in HK2 cells (Fig. 8c). Also, we observed that the expression levels of FN, SGLT2, and p-NF-КB p65 in HK2 cells decreased either in co-culture system of canagliflozin or MSCs for 72 h (Fig. 8d, e).

## Discussion

**Fig. 9:**
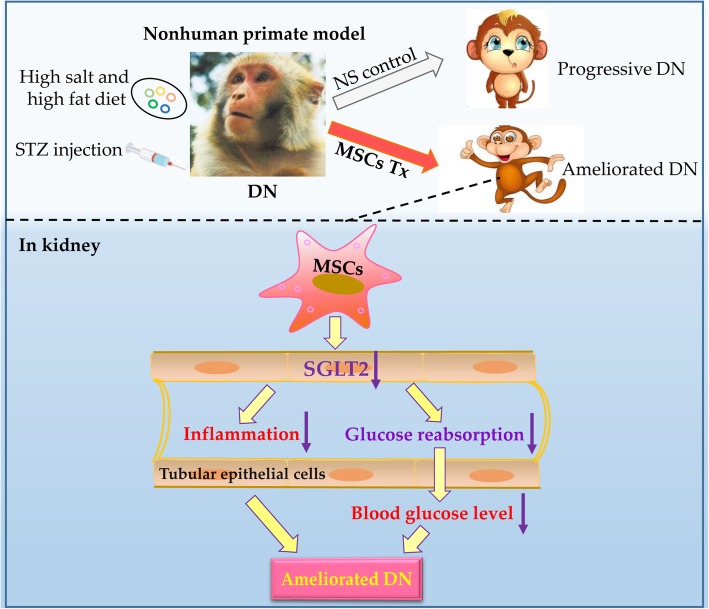
The hypothetical pathway through which MSC infusion ameliorate the development of DN. Preclinical nonhuman primate model of diabetic nephropathy (DN) was built by streptozotocin (STZ) injection and a high-salt and high-fat diet to rhesus macaque. Mesenchymal stem cells (MSCs) transplantation ameliorated diabetic nephropathy compared with normal saline (NS) infusion. The possible mechanisms were investigated by exploring the effects of MSCs on anti-inflammation and Na^+^-glucose cotransporter 2 (SGLT2) expression on tubular epithelial cells

For the first time, we explored the effects of MSCs on inflammation and SGLT2 expression in nonhuman primates with DN, and we also investigated the potential mechanisms of MSCs (Fig. 9). In our research, we developed a rhesus macaque model of DN and performed MSC transplantation four times over 2 months. By analyzing the labeled MSCs in organs, we determined that MSCs could migrate to the kidneys and play a role there. Because the expression levels of SGLT2 in renal sections of rhesus macaques who received MSCs were significantly lower than those who had NS injection, we speculated that MSCs had effects on regulating SGLT2 expression on renal tubular epithelial cells.

Although the improvement in glycemic control of DN rhesus macaques after MSC treatment may be explained by extra-renal effects, it also raises the interesting possibility that one reno-protective mechanism of MSC therapy is to inhibit diabetes-associated upregulation of SGTL2 in renal proximal tubular epithelial cells. Also, we observed reduced SGLT2 expression in renal sections and improved renal function after MSC transplantation, and these results were consistent with the previous findings in which the SGLT2 inhibitor was shown to rebalance tubuloglomerular feedback and improve eGFR [33]. On the basis of these observations, we speculate that the change of SGLT2 expression likely to be a result than a cause of MSCs, which strongly implicate that MSCs are more pleiotropic and better for clinical application than the use of SGLT2 inhibitor alone.

We explored the protective effects of MSCs on DN of nonhuman primates rather than small animals [34] and provided a basis for future preclinical research, which is essential for the clinical application of MSCs. MSCs inhibited SGLT2 expression and decreased the blood glucose level, ameliorated pathological changes in renal tissues, and reduced inflammation in the kidneys and blood circulatory system, which contributed to improved renal function. Our results were consistent with other studies that used inflammation as a therapeutic target and showed that decreased inflammation improved vascular function in kidney disease [35]. However, we provided new insights into anti-inflammation effects of MSCs potentially through inhibiting SGLT2 expression.

Diabetic nephropathy is a microvascular complication of diabetes, and we observed that MSC transplantation improved renal perfusion and clearance. Previously, we explored the effects of MSCs on endothelial cells and verified the protective effects of MSCs on high glucose- and palmitic acid-injured endothelial cells, which helped to demonstrate the functions of MSCs on blood vessels. In this study, we explored another important cell type in kidneys, tubular epithelial cells. Due to the different physiological characteristics between tubular epithelial cells and blood vessel endothelial cells, fatty acid would not directly interact with renal tubular epithelial cells. In addition, TNF-α has been demonstrated to indicate inflammation in DN [36]**.** Hence, under these conditions, a high level of glucose and palmitic acid does not fit to induce a damage model of renal tubular epithelial cells in this study; instead, glucose and rhTNF-α were proper to mimic the hyperglycemia and inflammatory microenvironment in kidneys. Both MSC and SGLT2 inhibitor canagliflozin decreased GT-induced inflammation of HK2 cells; however, co-culture of HK-2 and MSC under GT condition was associated with a striking induction of IL-6 in both cell types, which require further research about the potential effect and mechanism.

The main limitations of this study include that the high cost of nonhuman primates is an obstacle of exploring more information about the duration of MSCs that stayed at various organs. In addition, although we established the early stage of DN rhesus macaque model according to the standard diagnosis of human DN, which is featured by microalbuminuria and UACR in a range of 2.5–25 mg/mmol, the level of albuminuria in DN groups remained low throughout the experiment, which did not totally mimic the progressive changes of albuminuria with time in human DN. Although we analyzed the immune cells and vital signs of rhesus macaques after MSC transplantation, we did not measure the antibody levels of anti-human IgA, IgM, and IgG, so the immunogenicity of the human MSC in rhesus macaque is not fully determined.

## Conclusions

In conclusion, our study is an important step to explore the mechanism of MSCs in ameliorating the early stage of DN, which possibly through influencing SGLT2 expression and resulting in improved glycemic control and decreased inflammation. We hope our research provides a basis for inspiring new developments in the future.

## Additional file

Additional file 1: **Figure S1**. The surface markers and multiple differentiation potentialities of hUC-MSCs. a Flow cytometric analysis of cell markers of MSCs. b Osteogenic differentiation and adipogenic differentiation of MSCs were determined by alizarin red staining and Oil red O staining. Scale bar= 50 μm. **Figure S2**. The rhesus macaques tolerated the xenogeneic hUC-MSCs. a Flow cytometric analysis of the ratio of the CD4+/CD8+ cells. b Quantification of flow cytometric analysis. c Weight of rhesus macaque. d-f Sum and sort counting of lymphocytes (LYM) , monocytes (MONO), and neutrophils (NEU) in blood of rhesus macaques before and 1 week after normal saline or MSC transplantation. Each bar represents the mean±s.e.m., n≥3/group. * *p* < 0.05; # *p* < 0.05. **Figure S3**. Fluorescence microscopy of CM-Dil-labeled MSCs in rhesus macaques. Red fluorescence in organs of two rhesus macaques with diabetic nephropathy at 1 week after MSCs infusion. Scale bar (red) = 50 μm; scale bar (white) = 20 μm. Each bar represents the mean±s.e.m.. Four fields of each section, and 4 sections per rhesus macaque were observed and quantified. **Figure S4**. Contrast-enhanced ultrasound of the kidneys of rhesus macaques with MSC treatment. a Images of contrast-enhanced ultrasound (CEUS) of the kidney before and 1 month after MSC transplantation. b Analysis of the rise time (RT), mean transit time (MTT), time to peak (TTP), and time from peak to one half (TPH). c Quantification of the area under the descending curve (AUC). Each bar represents the mean±s.e.m., n=6. * *p* < 0.05. **Figure S5**. Effects of MSCs on HK2 cells at 72 hours after GT. a Western blot analysis of protein expression levels in GT-treated HK2 cells with and without MSC coculture. b Quantification of western blot analysis of protein expressions. c Effect of MSCs on the NO production ability in HK2 cells. d Levels of glucose in the culture medium of HK2 cells analyzed by the oxidase method. Each bar represents the mean±s.e.m., n≥3/group. * *p* < 0.05; ** *p* < 0.01; # *p* < 0.05. **Table S1**. Primary and secondary antibodies. **Table S2**. hUC-MSCs Quality Inspection report. **Table S3.** The list of primers sequences. (DOC 15731 kb)

## Data Availability

All data generated or analyzed during this study are included in this published article.

## Abbreviations

DN: Diabetic nephropathy
MSCs: Mesenchymal stem cells
NS: Normal saline
HK2: Proximal tubular epithelial cells
Scr: Serum creatinine
BUN: Blood urea nitrogen
eGFR: Estimated glomerular filtration rate
H&E: Hematoxylin and eosin
Masson: Masson’s trichrome
PAS: Periodic acid Schiff
TEM: Transmission electron microscopy
GBM: Glomerular basement membrane
SGLT2: Na^+^-glucose cotransporter 2
IL-6: Interleukin 6
TNF-α: Tumor necrosis factor alpha
Can: Canagliflozin
NF-КB: Nuclear factor-kappa B
