# Supplementary material for: Intervention for early diabetic nephropathy by mesenchymal stem cells in a preclinical nonhuman primate model
Source: Stem Cell Res Ther. 2019 Dec 2;10:363. doi: 10.1186/s13287-019-1401-z (PMC6889652; doi:10.1186/s13287-019-1401-z)
Supplement: Supplementary file 1 — Figure S1. The surface markers and multiple differentiation potentialities of hUC-MSCs. a Flow cytometric analysis of cell markers of MSCs. b Osteogenic differentiation and adipogenic differentiation of MSCs were determined by alizarin red staining and Oil red O staining. Scale bar= 50 μm. Figure S2. The rhesus macaques tolerated the xenogeneic hUC-MSCs. a Flow cytometric analysis of the ratio of the CD4+/CD8+ cells. b Quantification of flow cytometric analysis. c Weight of rhesus macaque. d-f Sum and sort counting of lymphocytes (LYM) , monocytes (MONO), and neutrophils (NEU) in blood of rhesus macaques before and 1 week after normal saline or MSC transplantation. Each bar represents the mean±s.e.m., n≥3/group. * p < 0.05; # p < 0.05. Figure S3. Fluorescence microscopy of CM-Dil-labeled MSCs in rhesus macaques. Red fluorescence in organs of two rhesus macaques with diabetic nephropathy at 1 week after MSCs infusion. Scale bar (red) = 50 μm; scale bar (white) = 20 μm. Each bar represents the mean±s.e.m.. Four fields of each section, and 4 sections per rhesus macaque were observed and quantified. Figure S4. Contrast-enhanced ultrasound of the kidneys of rhesus macaques with MSC treatment. a Images of contrast-enhanced ultrasound (CEUS) of the kidney before and 1 month after MSC transplantation. b Analysis of the rise time (RT), mean transit time (MTT), time to peak (TTP), and time from peak to one half (TPH). c Quantification of the area under the descending curve (AUC). Each bar represents the mean±s.e.m., n=6. * p < 0.05. Figure S5. Effects of MSCs on HK2 cells at 72 hours after GT. a Western blot analysis of protein expression levels in GT-treated HK2 cells with and without MSC coculture. b Quantification of western blot analysis of protein expressions. c Effect of MSCs on the NO production ability in HK2 cells. d Levels of glucose in the culture medium of HK2 cells analyzed by the oxidase method. Each bar represents the mean±s.e.m., n≥3/group. * p < 0.05; ** p < [file 13287_2019_1401_MOESM1_ESM.doc]

**Supplemental Materials and Methods**

**Color Doppler ultrasound and angiography**

Real-time Doppler ultrasound examination was performed using a Philips IU22 ultrasound machine. Anesthetized rhesus macaques were placed in the prone position for a general check of the renal parenchyma and intrarenal blood perfusion of the right and left kidneys. SonoVue (Bracco SpA, Milan, Italy) was used as an ultrasound contrast agent, which was intravenously administered by bolus (0.1 ml/kg) via the saphenous vein and blasted with flash after 5 mins. Percutaneous ultrasound-guided renal biopsy was performed in normal rhesus macaques and the macaques with diabetic nephropathy before and 1 month after MSC or NS infusion. Renal biopsy tissues were prepared for different experiments. Renal perfusion images were converted by QLAB quantification software and analyzed by an ultrasound doctor.

**The details of following methods:** Assessment of Cell Viability, Immunofluorescence**,** Flow cytometry, Western blotting**,** please refer to our previous research [1].

1. An X, Li L, Chen Y, Luo A, Ni Z, Liu J, et al. Mesenchymal Stem Cells Ameliorated Glucolipotoxicity in HUVECs through TSG-6. Int J Mol Sci. 2016;17:483.

**
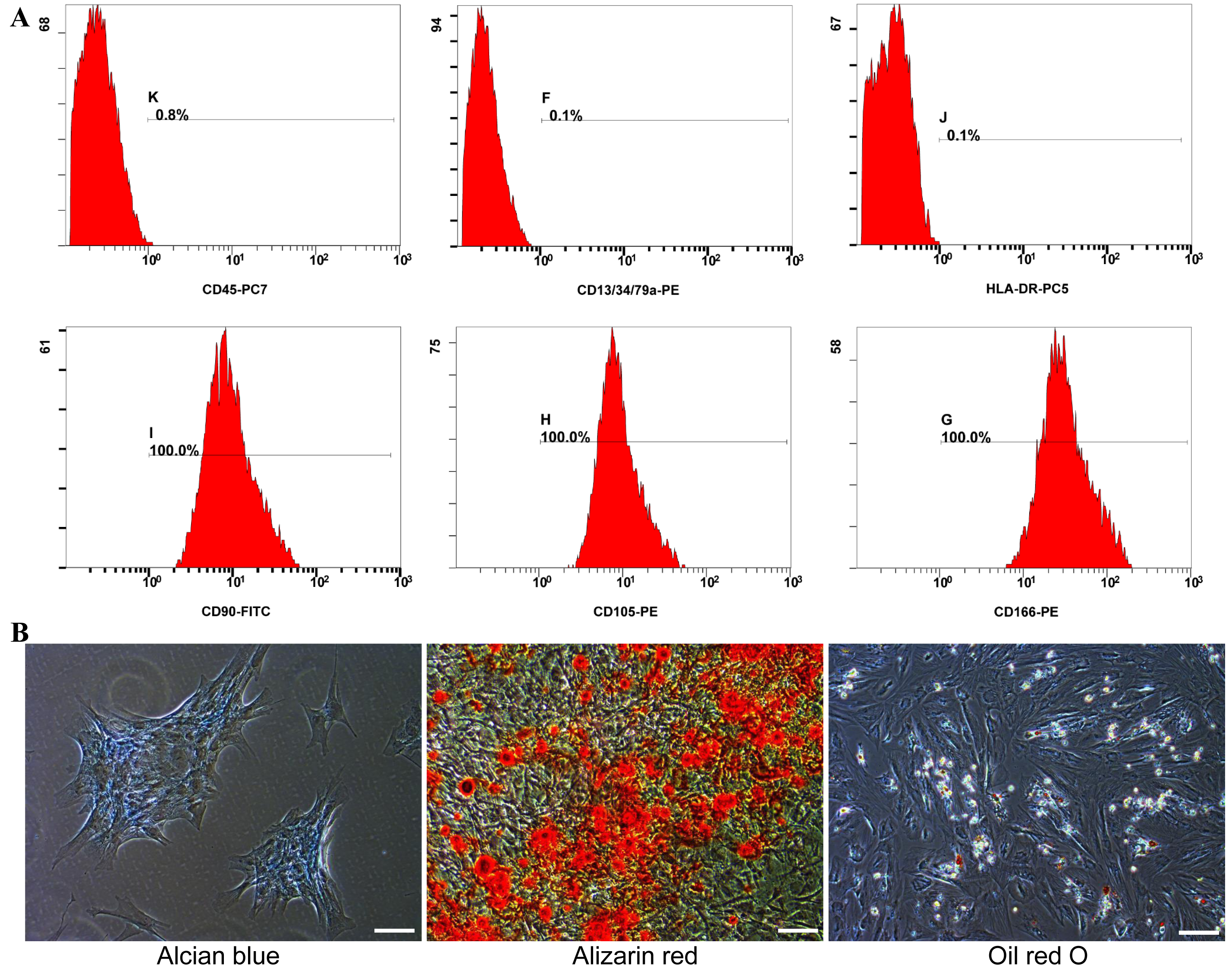
**

**Fig. S1. The surface markers and multiple differentiation potentialities of hUC-MSCs.** A: Flow cytometric analysis of cell markers of MSCs. B: Osteogenic differentiation and adipogenic differentiation of MSCs were determined by alizarin red staining and Oil red O staining. Scale bar= 50 μm.

**
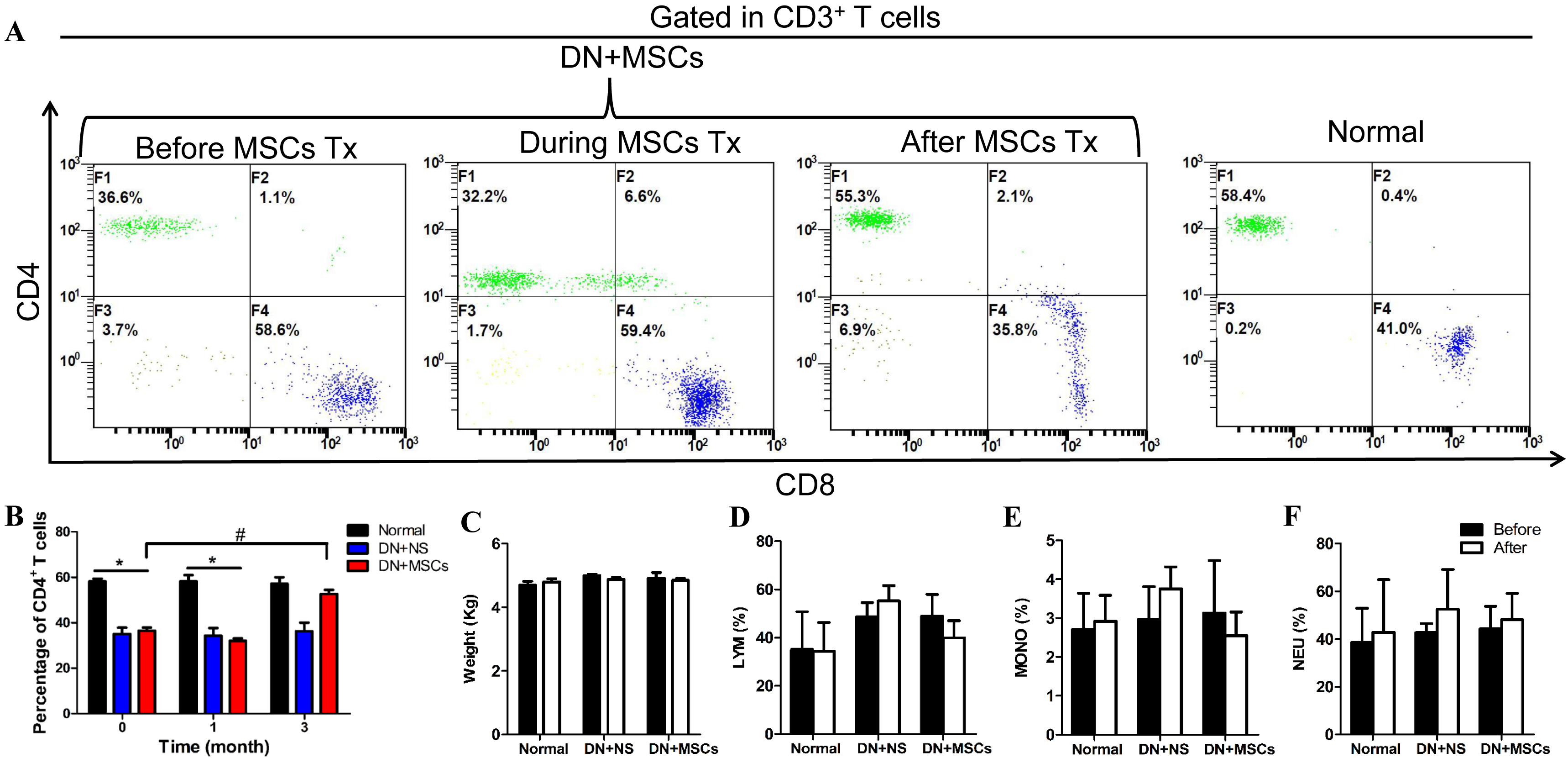
**

**Fig. S2. The rhesus macaques tolerated the xenogeneic hUC-MSCs**.

A: Flow cytometric analysis of the ratio of the CD4+/CD8+ cells. B: Quantification of flow cytometric analysis (A). C: Weight of rhesus macaque. D-F: Sum and sort counting of lymphocytes (LYM) , monocytes (MONO), and neutrophils (NEU) in blood of rhesus macaques before and 1 week after normal saline or MSC transplantation. Each bar represents the mean±s.e.m., n≥3/group. ** p* < 0.05; ***#*** *p* < 0.05.

**
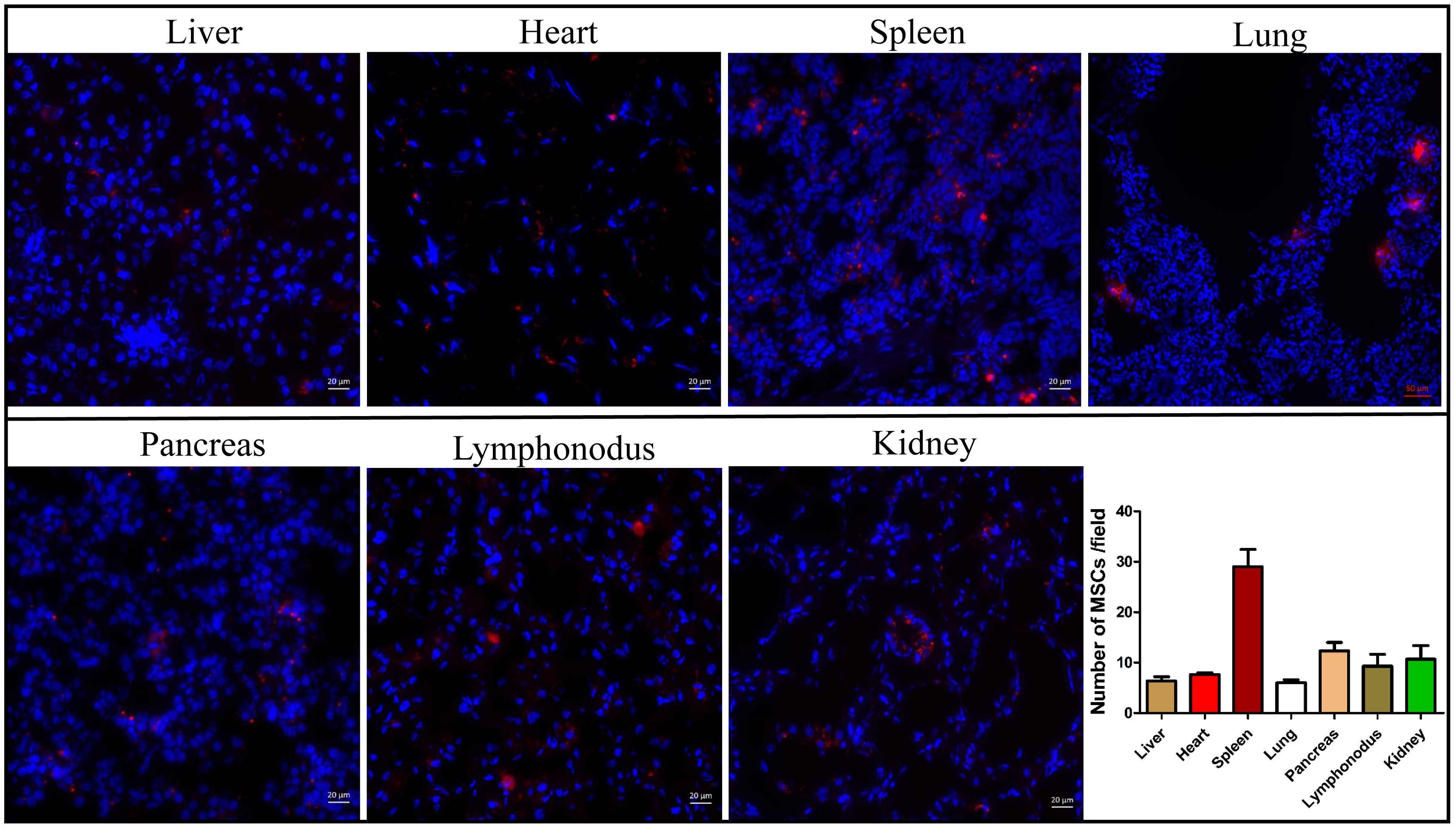
**

**Fig. S3.** **Fluorescence microscopy of CM-Dil-labeled MSCs in rhesus macaques.**

Red fluorescence in organs of two rhesus macaques with diabetic nephropathy at 1 week after MSCs infusion. Scale bar (red) = 50 μm; scale bar (white) = 20 μm. Each bar represents the mean±s.e.m.. Four fields of each section, and 4 sections per rhesus macaque were observed and quantified.


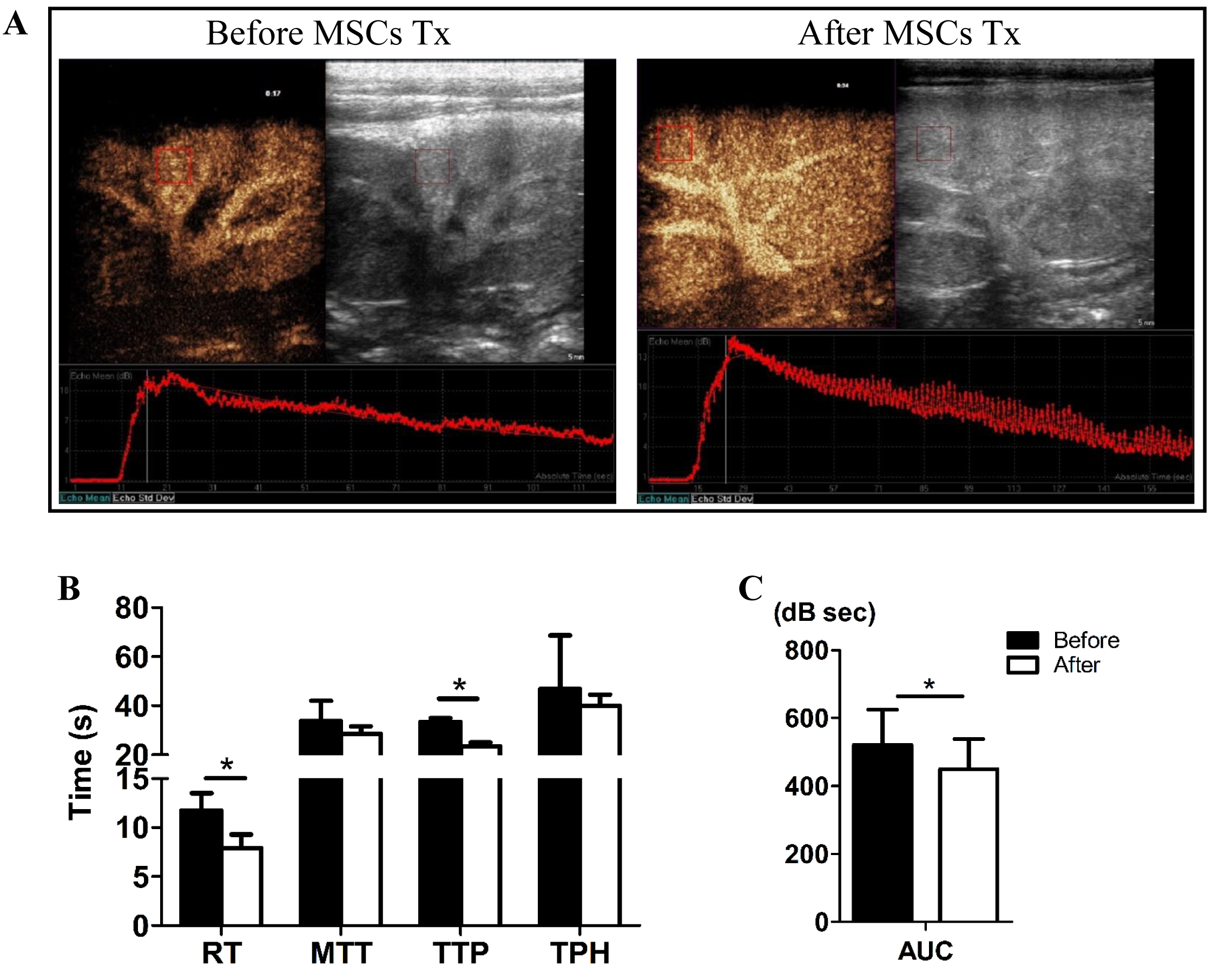


**Fig. S4. Contrast-enhanced ultrasound of the kidneys of rhesus macaques with MSC treatment.**

A: Images of contrast-enhanced ultrasound (CEUS) of the kidney before and 1 month after MSC transplantation. B: Analysis of the rise time (RT), mean transit time (MTT), time to peak (TTP), and time from peak to one half (TPH). C: Quantification of the area under the descending curve (AUC). Each bar represents the mean±s.e.m., n=6. ** p* < 0.05.

**
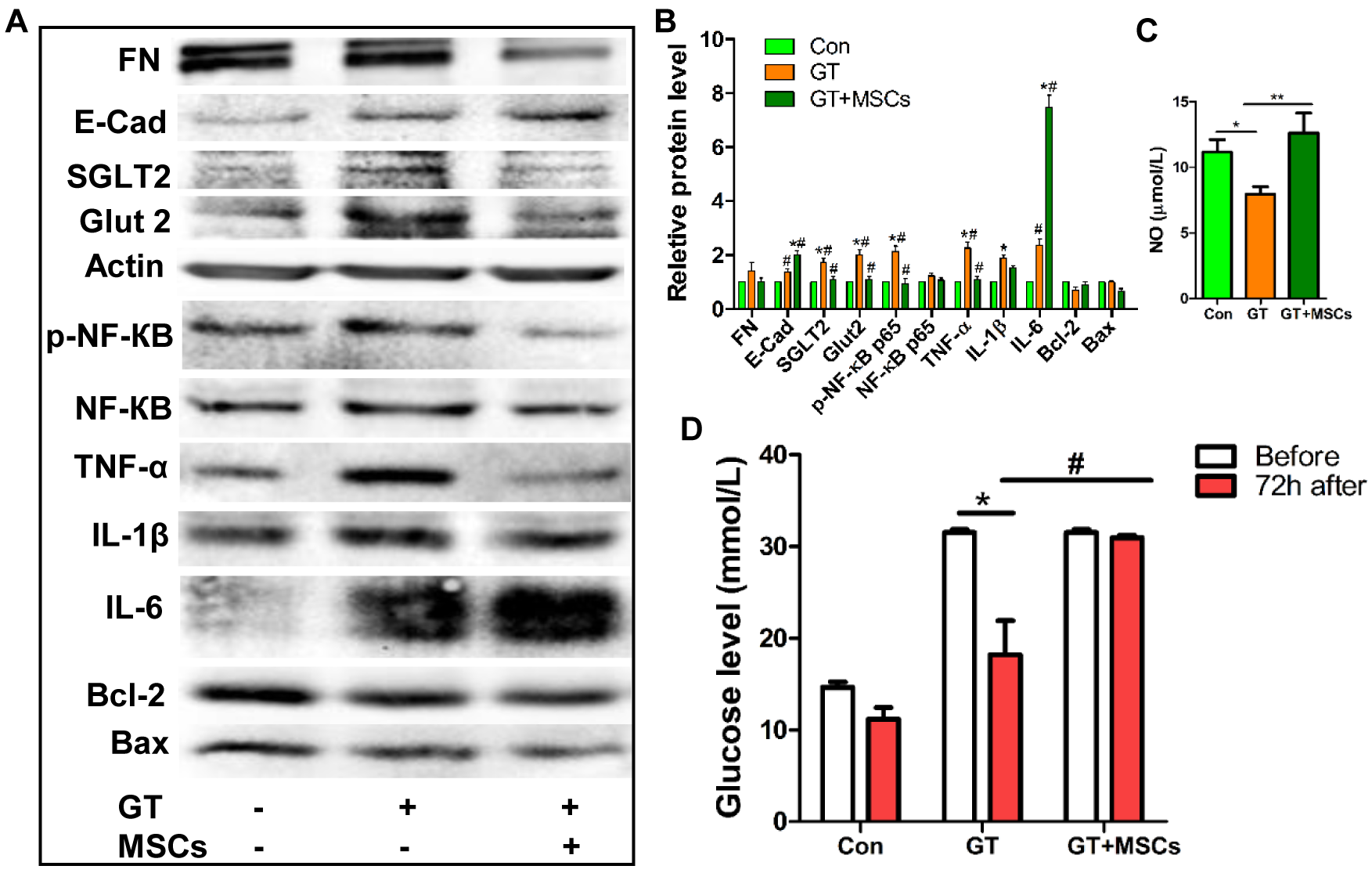
**

**Fig. S5. Effects of MSCs on HK2 cells at 72 hours after GT.**

A: Western blot analysis of protein expression levels in GT-treated HK2 cells with and without MSC coculture. B: Quantification of western blot analysis of protein expressions (A). C: Effect of MSCs on the NO production ability in HK2 cells. D: Levels of glucose in the culture medium of HK2 cells analyzed by the oxidase method. Each bar represents the mean±s.e.m., n≥3/group. ** p* < 0.05; *** p* < 0.01; ***#*** *p* < 0.05.

Table S1. Primary and secondary antibodies.

| Antibodies | Dilutions | Company |
| --- | --- | --- |
| Il-β | 1：200 (IHC), 1:1000 (WB) | Abcam |
| Il-6 | 1：100 (IHC), 1:1000 (WB) | Abcam |
| Il-16 | 1：400 (IHC), 1:1000 (WB) | Abcam |
| TNF-α | 1：400 (IHC), 1:1000 (WB) | Abcam |
| CTGF | 1：400 (IHC) | Abcam |
| SGLT-2 | 1: 200 (IF), 1：300 (IHC), 1:1000 (WB) | Abcam |
| MCP-1 | 1: 1000 (WB) | Abcam |
| E-cadherin | 1: 200 (IF), 1:500 (WB) | BD |
| FN | 1: 100 (IF), 1:500 (WB) | Proteintech |
| Alexa Fluor 488 goat anti-rabbit IgG (H+L) | 1: 500 (IF) | Invitrogen |
| Alexa Fluor 488 goat anti-mouse IgG (H+L) | 1: 500 (IF) | Invitrogen |
| β-Actin | 1:1000 (WB) | ABclonal |
| Glut-2 | 1:1000 (WB) | ABclonal |
| NF-КB p65 | 1:1000 (WB) | CST |
| phospho-NF-КB p65 | 1:1000 (WB) | CST |
| Bax | 1:1000 (WB) | CST |
| Bcl-2 | 1:1000 (WB) | CST |
| HRP Goat Anti-Rabbit IgG (H+L) | 1:2000 (WB) | ABclonal |
| HRP Goat Anti-Mouse IgG (H+L) | 1:2000 (WB) | ABclonal |

**Table S2.**

hUC-MSCs Quality Inspection report

|  | Category | Result |
| --- | --- | --- |
| Viral factors | HBsAg | Negative |
|  | Anti-HIV | Negative |
|  | Anti-HCV | Negative |
|  | Syphilis antibody | Negative |
|  | CMV antibody | Negative |
| Pathogens | Aerobic bacteria | Negative |
|  | Anaerobic bacteria | Negative |
|  | Fungi | Negative |
|  | Mycoplasma | Negative |
|  | Bacterial endotoxin | Qualified |

**Table S3. The list of primers sequences.**

Gene Forward Primer (5′ to 3′) Reverse Primer (5′ to 3′)

IL-1β GCCGTGTCAGTTGTTGTAGC TGAAGGGAATCAAGGTGCTC

IL-6 TACATCCTCGACGGCATCTC GCCATCTTTGGAAGGTTCAG

IL-8 ACTCCAAACCTTTCCACCC AACTTCTCCACAACCCTCTGC

IL-10 AGCTGAGAACCAAGACCCAGAC GAAATCGATGACAGCGCCGTAG

TNF-α CTGCCTGCTGCACTTTGGA TTGAAGAGGACCTGGGAGTAGAT

TGF-β CCGACTACTACGCCAAGGA GAGAGCAACACAGGTTCAGG

MCP-1 AGCCAGATGCAATCAATGCC GGGTCAGCACAGATCTCCTT

CCL-5 CCTGCTGCTTTGCCTACATT GCACACACTTGGCGATTCT

SGLT2 GTCTTCAGTCTCCGGCATAGCA CCTGGGGCTCATTCATCTCCAT

GAPDH GAACGGGAAGCTCACTGG GCCTGCTTCACCACCTTCT

β-Actin CCACGAAACTACCTTCAACTCC GTGATCTCCTTCTGCATCCTGT
